# Effects of 12-Week Supplementation with Coffee Diterpene Cafestol in Healthy Subjects with Increased Waist Circumference: A Randomized, Placebo-Controlled Trial

**DOI:** 10.3390/nu16193232

**Published:** 2024-09-24

**Authors:** Fredrik D. Mellbye, Mi D. Nguyen, Kjeld Hermansen, Per B. Jeppesen, Zheer K. Al-Mashhadi, Steffen Ringgaard, Søren Gregersen

**Affiliations:** 1 Steno Diabetes Center Aarhus, Aarhus University Hospital, 8200 Aarhus, Denmark; mi-nguyen@hotmail.com (M.D.N.); zhehus@rm.dk (Z.K.A.-M.); soeren.gregersen@aarhus.rm.dk (S.G.); 2 Department of Endocrinology and Internal Medicine, Aarhus University Hospital, 8200 Aarhus, Denmark; kjeld.hermansen@dadlnet.dk (K.H.); per.bendix.jeppesen@clin.au.dk (P.B.J.); 3 Department of Clinical Medicine, Aarhus University, 8200 Aarhus, Denmark; 4 The MR Research Centre, Aarhus University, 8200 Aarhus, Denmark; steffen@clin.au.dk

**Keywords:** coffee, diabetes, obesity, cafestol, glucose, placebo, metabolism

## Abstract

*Background*: Coffee consumption is inversely associated with type 2 diabetes. Cafestol, a bioactive compound in coffee, has demonstrated glucose-lowering and insulin-secretory properties in cell and animal studies. The acute effects of cafestol on glucose metabolism in humans have only been briefly investigated, and longer-term effects have not been explored. This study aimed to assess the effects of purified cafestol on insulin sensitivity and other metabolic parameters in healthy individuals with increased waist circumference at risk of developing type 2 diabetes. *Methods*: A 12-week randomized, placebo-controlled, parallel trial was conducted with 40 participants. Insulin suppression tests, mixed meal tests, and MRI scans were performed before and after the intervention. *Results*: Administering 6 mg of cafestol twice daily did not alter insulin sensitivity or glucose tolerance but led to significant reductions in body weight (2%), visceral fat volume (5%), and gamma-glutamyl transferase levels (15%) compared to the placebo. *Conclusions*: Cafestol may hold promise for weight and visceral fat reduction. Cafestol did not improve insulin sensitivity or glucose tolerance in this study but might still contribute to the observed inverse association between coffee consumption and type 2 diabetes. Future research should explore higher dosages and longer treatment durations, particularly in individuals with impaired glucose metabolism and type 2 diabetes.

## 1. Introduction

According to the International Diabetes Federation, the global number of adults living with diabetes reached 537 million in 2021 [1]. It is crucial to discover new, safe, and cost-effective approaches to prevent and treat type 2 diabetes [2]. Coffee consumption is inversely associated with type 2 diabetes, demonstrating a 25–29% risk reduction in individuals who consume four cups of coffee daily compared to one cup or no coffee [3,4,5]. However, the causal evidence regarding the beneficial effects of coffee consumption on surrogate markers for type 2 diabetes development is not known. A 6-month placebo-controlled randomized trial in insulin-resistant overweight subjects of Alperet et al. [6] using instant coffee and a coffee-tasting placebo beverage did not find improvements in insulin sensitivity measured by a hyperinsulinemic-euglycemic clamp. Furthermore, compared to no coffee consumption, an 8t-week instant coffee intervention in overweight subjects did not change the area under the curve of glucose during an oral glucose tolerance test (OGTT) [7], while a 16-week intervention in overweight men with mild-to-moderately elevated fasting glucose using instant coffee showed modest reductions in the glucose response during OGTT [8]. Most coffee intervention studies use instant coffee, which only contains negligible amounts of diterpenes cafestol and kahweol [9,10]. Cafestol is a lipophilic substance present in boiled, Turkish, French press, and espresso coffee and is most known for its LDL-cholesterol-raising effects [9,10]. In previous in vitro studies, we observed that cafestol acutely increases insulin secretion from INS-1E rat clonal beta cells and enhances glucose uptake in a human skeletal SkMC C-12580 muscle cell line [11]. Additionally, in a 10-week cafestol intervention study [12] in KKAy mice, a diabetic mouse model [13], cafestol resulted in 30% lower glucose levels, improved insulin sensitivity, and increased insulin secretory capacity from isolated islets of Langerhans [12]. More recently, we studied the acute effects of cafestol and kahweol in participants with a large waist circumference [14] and participants with type 2 diabetes [15]. We demonstrated that these compounds appear to acutely and briefly lower glucose, particularly in individuals with impaired glucose tolerance and/or impaired fasting glucose or type 2 diabetes. Several studies have explored the long-term effects of coffee diterpenes on serum lipids and transaminases using coffee oils or unfiltered coffee brews with high cafestol content [16,17,18,19,20,21]; however, their effects on glucose metabolism, fat mass, and the potential of coffee diterpenes as a preventive measure against type 2 diabetes have not previously been thoroughly investigated.

The objective of the present study is to investigate the effects of a 12-week dietary supplementation with cafestol on insulin sensitivity, mixed-meal responses, body fat distribution, and selected other metabolic biomarkers in healthy participants with a large waist circumference. We hypothesize that a 12-week cafestol dietary supplementation will improve insulin sensitivity, the glucose response curve following a mixed meal, the fat mass, and selected other metabolic biomarkers.

## 2. Materials and Methods

### 2.1. Trial Design

This was a 1:1 randomized, double-blinded, placebo-controlled, parallel-group study conducted at Steno Diabetes Center Aarhus, Denmark. Participants were randomly assigned to one of two parallel groups, receiving either 6 mg cafestol or placebo capsules twice daily for 12 weeks. The study comprised a total of six visits. Prior to the first visit, participants were interviewed by phone or email and provided with informational material.

### 2.2. Visits

At the first visit, subjects were requested to sign the consent form to participate. Once inclusion and exclusion criteria were confirmed, a blinded continuous glucose monitor (CGM) sensor was placed on the upper arm. Additionally, a 24-h ambulatory blood pressure monitor (ABPM) was mounted on the other arm and worn for the next 24 h. Participants returned for visit 2 after a minimum of 7 days. During the second visit, participants underwent a mixed meal test (MMT) and a magnetic resonance imaging (MRI) scan, and fasting blood samples were drawn. During the third visit, participants underwent an insulin suppression test (IST). Following this visit, the 12-week cafestol/placebo intervention was initiated. After 6 weeks of intervention, liver and kidney function were assessed with blood samples. Visits 4 (10–11 weeks after visit 3), 5, and 6 were repeats of visits 1, 2, and 3, respectively.

### 2.3. Tests

#### 2.3.1. MMT

The MMT was performed following an eight-hour overnight fast (water allowed). A retrograde peripheral venous catheter was placed in the median cubital vein, and blood samples were collected at specified time points (−15, 0, 15, 30, 60, 90, 120, 180 and 240 min). The participants consumed a meal consisting of 75 g of white bread, 10 g of butter, 30 g of cheese, and 200 mL of orange juice at time point 0 min. The meal’s total energy content was 412 kcal, composed of 15.8 g protein (16 E%), 13.6 g fat (30 E%), and 55.1 g carbohydrate (54 E%).

#### 2.3.2. IST

We performed a modified two-stage IST [22] to assess insulin sensitivity. At stage 2, this method yields a steady-state plasma glucose (SSPG) level closely associated with the M-value obtained from the gold-standard hyperinsulinemic-euglycemic clamp [23]. An SSPG is also reached at stage 1, simulating fasting physiological conditions. At this stage, the steady state level of free fatty acids (FFA) reflects suppression of lipolysis and, hence, fat-tissue insulin sensitivity [22]. The IST was performed after only an eight-hour overnight fast, but otherwise, as described by McLaughlin [22].

#### 2.3.3. MRI Scan

MRI examinations were performed using a 3T Siemens Skyra MR scanner with multi-element posterior and anterior coils used for signal reception. To assess lipid volume, a 3-stack fast gradient echo 3D Dixon imaging sequence was utilized. Axial slices were acquired, covering the abdomen from the top of the liver to mid-thighs, with a slice thickness of 3 mm, no gap, and an acquired pixel size of 1.6 mm × 2.1 mm. The repetition time (TR) and echo time (TE) were 3.97 and 1.23 ms, respectively, and images were acquired over three breath holds. Liver fat fraction was assessed through single voxel PRESS spectroscopy during free breathing. The voxel was 3 mm × 2 mm × 2 mm and was positioned in either segment VI or VII of the liver. TR and TE were set to 3000 ms and 33 ms, respectively, and 80 averages were acquired with and without water suppression to quantify the fat-to-water ratio. Using the Dixon fat images, subcutaneous and visceral fat volumes were calculated. The fat in all images from the top of the liver to the femur head was segmented into the two domains using a semi-automatic method based partially on thresholding.

#### 2.3.4. ABPM

Participants were equipped with a blinded Spacelabs OnTrak blood pressure monitor (Spacelabs Healthcare, Snoqualmie, WA, USA) for 24-h. Blood pressure data were processed using Spacelabs Sentinel 11 software.

#### 2.3.5. CGM

Participants wore blinded FreeStyle Libre Pro IQ 1.4.1 CGM sensors (Abbott Laboratories, Abbott Park, IL, USA). The glucose range used for time in range calculation was 3.9–10 mmol/L [24]. Data was collected with FreeStyle Libre Pro Software version 1.0, from which raw data were exported for later analysis.

### 2.4. Participants

Eligible participants were adults between 18 years and 80 years with a waist circumference > 102 cm (men) or 88 cm (women) and, therefore, at risk of developing type 2 diabetes [25]. Exclusion criteria were type 2 diabetes diagnosis, HbA1c > 6.5% (48 mmol/mol) or treatment with antidiabetic drugs, pregnancy, planned pregnancy, breastfeeding and/or significant comorbidity.

The study was conducted at Steno Diabetes Center Aarhus, Aarhus University Hospital. Study personnel were the same physician and the same laboratory technicians on all study days, with intermittent additional assistance from a medical student and another physician on some study days when needed. Participants were recruited through advertisements online, in newspapers, and through official hospital social media posts.

### 2.5. Interventions

Participants self-administered capsules with 6 mg cafestol or placebo twice daily at breakfast and dinner. The cafestol and placebo capsules were assembled, labeled, and sealed in capsule containers by the pharmacy Glostrup Apotek (Glostrup, Denmark). Cafestol was purified (99%) from coffee beans by PhytoLab GmbH & Co. KG (Vestenbergsgreuth, Germany). Participants were allowed to drink unlimited amounts of paper-filtered drip coffee and instant coffee in which the diterpene amounts were negligible [10] but were limited to only one unfiltered coffee beverage (French press coffee, boiled coffee, espresso) per day throughout the entire study. Adherence to these restrictions was assessed with a questionnaire at visit 6.

### 2.6. Outcomes

Outcomes were measured before and after intervention. Primary outcomes were SSPG at stage 2 (time point 220–240 min) and at stage 1 (time point 100–120 min) during IST, and 4-h total area under the curve (tAUC) for glucose during MMT. Secondary outcomes were 4-h tAUC for insulin, triglycerides (TG), and glucagon during MMT, HbA1c, liver fat content measured by MR spectroscopy, visceral and subcutaneous fat volume measured with MRI Dixon scans, systolic and diastolic blood pressure when awake and asleep, mean-glucose, time in range, and glucose variability measured with CGM and selected fasting blood samples.

### 2.7. Ancillary Outcomes

FFA levels during the IST were analyzed but not prespecified in the protocol. Psoas major and Gluteus Maximus muscle fat content was also analyzed but not prespecified in the protocol. Weight and BMI were prespecified in the protocol and necessary to measure to calculate infusion rates but were not originally listed as outcomes.

### 2.8. Sample Size

To detect a significant 10% reduction in the glucose tAUC after an MMT (SD ± 10% of anticipated mean), with a power of 80% and a two-sided 5% significance level, we determined that a sample size of 40 participants would be necessary, yielding 16 participants in each group given an anticipated dropout rate of 20%. To detect a 20% decrease in stage 2 SSPG, an effect seen after modest weight loss [26], only 14 participants in each group were needed with the same alpha, beta, and power levels. We did not have sufficient data to perform a power calculation for change in stage 1 SSPG. McLaughlin et al. found stage 1 SSPG levels of 6.5 (SD ± 1.6) and 4.9 (SD ± 1.1) mmol/L in insulin-resistant and sensitive non-diabetic overweight/obese women, respectively [22].

### 2.9. Randomization

Independent pharmacists from the pharmacy Glostrup Apotek generated a list of numbers 1–40 and assigned treatment by drawing cafestol (20) or placebo (20) without replacement. They then produced capsule containers with identical labels, each marked with a unique number between 1 and 40. The first participant enrolled in the study received the capsule container marked as number 1, the second participant received container number 2, and so on.

### 2.10. Blinding

Participants and study personnel (study physician, laboratory technician, medical student) were blinded from the treatment allocation. The cafestol and placebo capsules were indistinguishable in appearance. Only independent pharmacists had access to the allocation list, which was securely kept at the pharmacy. The list was revealed to the study physician after the study, initial data analysis, and outcome assessment were completed.

### 2.11. Statistical Analysis

All statistical analyses were performed in R version 4.2.1. Total AUCs were calculated using the trapezoid method. Glucose levels at IST start and at steady state (Stage 1 and Stage 2), FFA, glucagon, and insulin were calculated individually for each participant on both ISTs separately as participant-mean levels at time points −15 and 0 min; 100, 110, and 120 min; and 220, 230 and 240 min, respectively. MR spectroscopy spectra were analyzed using LCModel version 6.3-1L. MRI Dixon-scan fat content was assessed using locally developed software called Siswin version 0.95. Baseline and post-intervention mean or median results are found in Supplementary Table S1. Within-group comparisons are made with paired t-tests on means or Wilcoxon-signed rank tests on medians. Between-group outcome differences after intervention were evaluated using one-way ANCOVA adjusting for baseline results and sex. A generalized linear model on, depending on whether relevant assumptions were met or not, normal and log-transformed data rendered absolute β_cafestol_ and relative exp(β_cafestol_) values denoting the absolute or relative difference between groups, respectively. In instances where post-intervention data was missing, the corresponding pre-intervention measurement from the same participant was removed, and vice versa. A detailed description of the statistical analysis is found in the Appendix A.

### 2.12. Registration

The study is registered at ClinicalTrials.gov with identifier NCT05672433.

## 3. Results

### 3.1. Participant Flow

**Figure 1:**
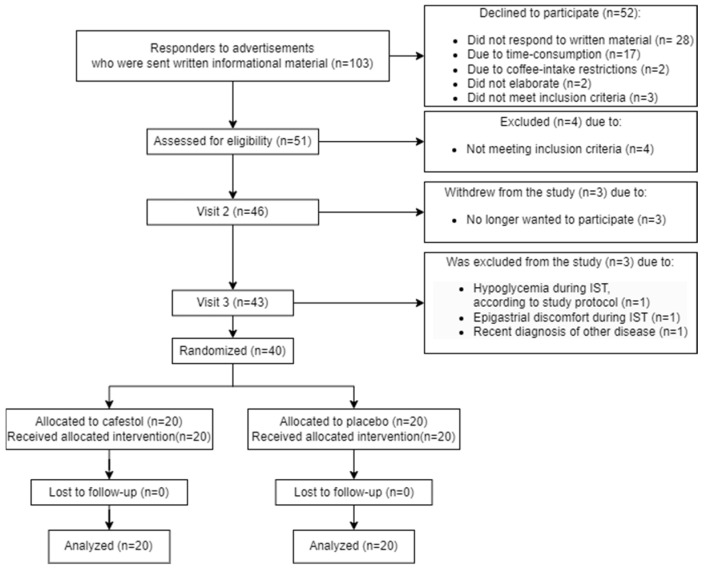
Participant flow diagram.

A detailed participant flow diagram is found in Figure 1.

### 3.2. Recruitment

The study timeline started with the first newspaper advertisement on 23 April 2022, followed by the first screening on 30 May 2022. The first 12-week cafestol/placebo intervention began on 22 June 2022, with subsequent participants being screened and initiated continuously. The study concluded with the last participant’s final visit on 22 December 2022.

### 3.3. Baseline Data

Baseline data are listed in Table 1.

### 3.4. Outcomes

Between-group differences are listed in Table 2 and Table 3. Within-group differences are listed in Supplementary Table S1.

#### 3.4.1. Primary Outcomes

**Figure 2:**
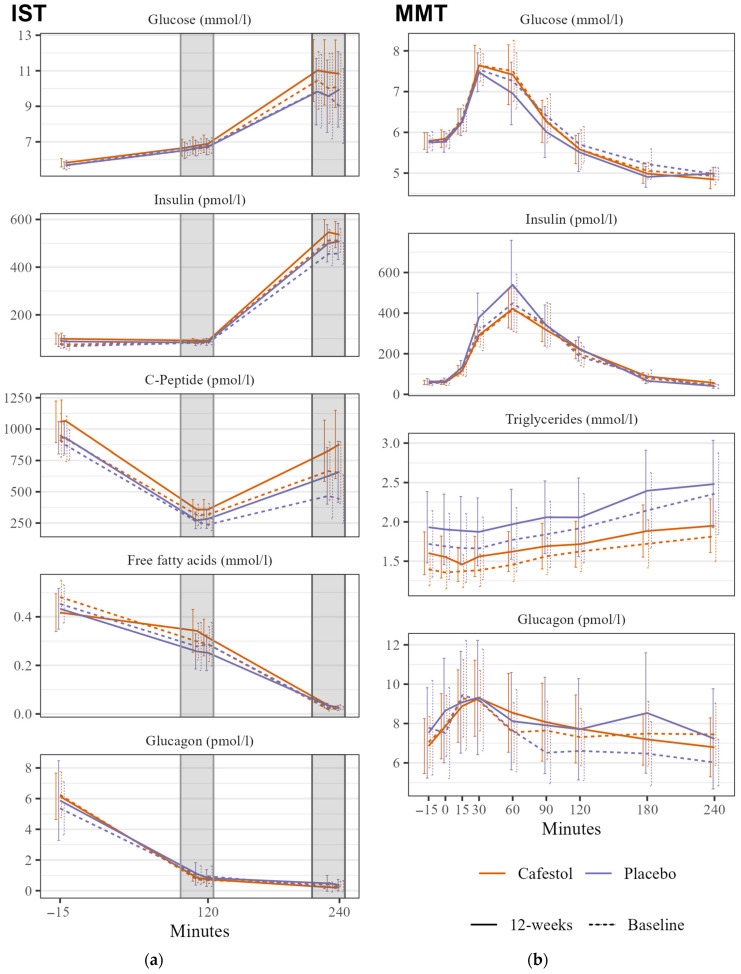
Insulin Suppression Test (IST) and Mixed Meal Test (MMT) in 20 subjects receiving cafestol (orange) and 20 subjects receiving placebo (blue) before (dashed line) and after (solid line) intervention. (**a**) IST: Mean glucose, insulin, c-peptide, FFA, and glucagon levels during insulin suppression tests before infusions, at stage 1 (100–120 min) and stage 2 (220–240 min) before intervention (dashed line) and after the 12-week intervention (solid line). (**b**) MMT: Glucose, insulin, triglyceride, and glucagon curves during mixed meal test before intervention (dashed line) and after the 12-week intervention (solid line). Error bars represent 95% CI.

There were no significant changes in SSPG during IST within or between the groups. There was no significant between-group difference in mixed meal glucose tAUC (Figure 2).

#### 3.4.2. Secondary Outcomes

There was no significant between-group HbA1c difference. There were between-group differences with lower mixed meal glucagon and insulin tAUCs in the cafestol group, but they were statistically insignificant. TG tAUC changes did not differ between treatment groups (Figure 2). There were no between-group differences in any CGM outcome, HOMA-IR, or blood pressure. Mean visceral fat volume was significantly reduced by 400 mL within the cafestol group (*p* = 0.01), and there was a significant difference in volume reduction between the treatment groups with a β_cafestol_ of −0.39 (95% CI −0.7–−0.1, *p* = 0.014). Subcutaneous fat volume was not altered after each intervention. There was no absolute between-group difference in liver fat content (*p* = 0.9). Gamma-glutamyl transferase was significantly lowered after cafestol intake compared to placebo. There were no between-group differences in LDL-cholesterol. The remaining between-group differences in fasting blood samples are presented in Table 3.

#### 3.4.3. Ancillary Analyses

There was no significant between-group HbA1c difference. There were between-group differences with lower mixed meal glucagon and insulin tAUCs in the cafestol group, but they were statistically insignificant. TG tAUC changes did not differ between treatment groups (Figure 2). There were no between-group differences in any CGM outcome, HOMA-IR, or blood pressure. Mean visceral fat volume was significantly reduced by 400 mL within the cafestol group (*p* = 0.01), and there was a significant difference in volume reduction between the treatment groups with a β_cafestol_ of −0.39 (95% CI −0.7–−0.1, *p* = 0.014). Subcutaneous fat volume was not altered after each intervention. There was no absolute between-group difference in liver fat content (*p* = 0.9). Gamma-glutamyl transferase was significantly lowered after cafestol intake compared to placebo. There were no between-group differences in LDL-cholesterol. The remaining between-group differences in fasting blood samples are presented in Table 3.

### 3.5. Harms

At study completion, six participants believed they received cafestol (of whom two received cafestol), ten participants believed they received a placebo (of whom seven received a placebo), and the last 24 participants had no opinion. Six participants in total reported discomfort from capsules (four in the placebo group, two in cafestol group); five participants reported increased flatulence and/or loose stools, and one participant experienced a sore throat the first two weeks of treatment (placebo). Seven participants reported discomfort during study days, with mild complaints of headaches, loose stools and/or nausea. No participant had alarming liver- or kidney function changes measured with blood samples after six weeks and at the study’s end. One participant in the cafestol group experienced a slight increase in alanine aminotransferase at the study’s end due to infectious mononucleosis diagnosed by the primary healthcare provider. Only two participants, one in each group, did not adhere to unfiltered coffee consumption restrictions. The subject in the placebo group reported drinking three cups of espresso daily, and the participant in the cafestol group reported drinking five cups of French press coffee per day.

## 4. Discussion

This study represents the first longer-term human intervention in healthy subjects with increased waist circumference comparing purified cafestol to placebo. All participants, randomly assigned to either the cafestol or placebo group, successfully completed the 12-week intervention without experiencing any significant adverse effects from capsule consumption. A modest dosage of 6 mg of cafestol, administered twice daily, was employed, and the total 12 mg cafestol is approximately equivalent to the cafestol content found in four 150-mL cups of French press coffee or 2–3 cups of Turkish- or boiled coffee [9,10]. Interestingly, we found that a 12-week cafestol intervention reduces body weight, visceral fat volume, and gamma-glutamyl transferase levels compared to the placebo. However, we found no effect of cafestol on insulin sensitivity, mixed meal glucose, insulin, TG, and glucagon response curves; CGM data; diurnal blood pressure; or fasting blood samples such as HbA1c and HOMA-IR. It is noteworthy that we did not find any impact of cafestol on LDL-cholesterol levels.

We did not observe any improvement in insulin sensitivity during the hypoinsulinemic stage 1 or hyperinsulinemic stage 2 of the IST. This lack of effect may indicate that cafestol does not enhance insulin sensitivity, or it could be attributed to factors such as a cafestol dosage that is too low, insufficient treatment duration, or a combination thereof. In retrospect, now that the safety of the given treatment has been established, a higher dosage could have been preferable. However, since this study represents the first long-term intervention involving purified cafestol in humans, caution was exercised. Future investigations should consider employing higher dosages of cafestol to elucidate its effects on insulin sensitivity and other cardiometabolic parameters in prediabetic and type 2 diabetes subjects.

Significant between-group differences in free fatty acid (FFA) levels were observed during the ISTs. Following treatment, the cafestol group exhibited higher FFA levels during the hypoinsulinemic stage 1. In the hyperinsulinemic stage 2, FFA release was adequately suppressed in both groups. In a study of insulin-resistant (*n* = 28) and insulin-sensitive (*n* = 28) women with overweight or moderate obesity, there was a clear between-group difference in FFA levels during stage 1 of the IST caused by an insufficient suppression of lipolysis in the insulin resistant group, and the authors claim the stage 1 FFA to be more informative than stage 1 SSPG [22]. Thus, the IST stage 1 FFA levels found in this study could suggest an increase in insulin resistance in adipose tissue after cafestol intervention.

There was no significant between-group difference in glucose tAUC during the MMT. In our previous studies, we observed an acute decrease in glucose levels during oral glucose tolerance tests when cafestol and kahweol were consumed alongside the glucose load. However, the absence of a similar effect in this study could potentially be attributed to the lower dosage used, the absence of kahweol, or the possibility that the glucose-lowering effects of these diterpenes are mediated acutely rather than during longer-term ingestion.

There was no reduction in HbA1c, and there was no significant between-group difference. These results are surprising, especially considering both groups had lower mean glucose levels after the interventions measured with CGM.

After cafestol treatment, the visceral fat volume was significantly reduced by 400 mL compared to the placebo group. Additionally, a significant between-group difference in weight/BMI change was observed, with the cafestol group experiencing weight loss accounting 880 g, while the placebo group exhibited weight gain of 920 g. Interestingly, cafestol treatment also reduced gamma-glutamyl transferase levels compared to placebo. Elevated gamma-glutamyl transferase levels are associated with a greater risk of type 2 diabetes [27,28,29], and reduction of gamma-glutamyl transferase after boiled coffee consumption has previously been reported [30]. There were no absolute mean changes in liver fat content. It may be that the dosage and duration of the cafestol intervention was adequate to promote weight loss and reduction in visceral fat volume but insufficient to significantly improve glucose tolerance or insulin resistance as measured in the ISTs and MMTs. In a study on weight loss and insulin resistance, a 9–10% weight loss resulted in an IST SSPG reduction of 30% [31]. Green coffee extract has been found to reduce BMI, especially in overweight or obese subjects [32]. Others claim that coffee decreases body weight and visceral fat through adipocyte cell cycle regulation, affecting transcription factors and lipogenesis-related proteins and that coffee alters gut microbiota [33,34]. Remarkably, Alperet et al. also found that a 6-month coffee intervention reduced fat mass by 3.7% and reduced weight by 1%, without altered fasting glucose or insulin sensitivity, compared to placebo [6]. However, the reduction in weight and fat mass observed in that study is unlikely to be attributed to cafestol, considering that diterpene-scant instant coffee was used. Nonetheless, our novel findings of reduced visceral fat volume, decreased body weight, and reduced gamma-glutamyl transferase in humans following cafestol treatment compared to placebo hold significant implications and warrant further investigation.

Cafestol has previously been shown to elevate LDL-cholesterol, with each 10 mg increase in cafestol being associated with a linear rise of 0.13 mmol/L in cholesterol, predominantly due to LDL-cholesterol elevation [35]. However, in this study, the dosage employed did not lead to elevated LDL-cholesterol or total cholesterol levels, nor did it raise systolic or diastolic blood pressure. Therefore, it can be concluded that the dosage used in this study is unlikely to pose a cardiovascular risk.

Participants were instructed to limit their consumption of unfiltered coffee to one cup per day while unrestrictedly consuming filtered coffee, irrespective of their coffee intake patterns prior to the study. Consequently, habitual unfiltered coffee consumers in the placebo group and heavy consumers (more than four unfiltered cups per day) in the cafestol group were exposed to a lower cafestol intake during the intervention period compared to their pre-study habits, and we have thus examined both the effects of cafestol but also in some participants the effects of removing or reducing daily cafestol consumption. Unfortunately, measurement of cafestol content in blood samples was not feasible; however, findings from a study in subjects with ileostomy suggest an approximate absorption rate of 70% in the small intestines [17].

It should be noted that the development of type 2 diabetes can occur gradually, and a 12-week intervention period, not remotely close to the decades-long beneficial coffee exposure reported in epidemiological literature, may not be sufficient to produce significant improvements in insulin sensitivity and glucose tolerance.

We employed specific inclusion criteria, focusing on individuals with a large waist circumference, as they are at an elevated risk of developing type 2 diabetes. The study encompassed a diverse range of healthy participants, including both males and females, spanning a wide age range of 25 to 78 years. Therefore, the findings of our study hold generalizability for a broad spectrum of adults exhibiting increased waist circumference in a Danish population. However, despite meeting the inclusion criteria, a significant proportion of the study population was not particularly insulin resistant, as evidenced by low HbA1c levels, CGM mean glucose measurements, SSPG during ISTs, and normal MMT glucose response curves. As a result, the potential for improvement in our measured outcomes may have been limited in these participants. Instead, more pronounced effects may been seen in a population of subjects with type 2 diabetes.

Furthermore, we employed a random allocation procedure to assign participants to either the treatment or placebo groups without specifically matching for factors such as sex, age, or baseline HbA1c levels. As a result of chance, dissimilarities in baseline characteristics were observed between the cafestol and placebo groups, including a higher proportion of younger participants with a lower degree of insulin resistance in the placebo group. Moreover, the placebo group exhibited a higher female-to-male ratio. Although implementing block randomization procedures might have ensured more homogenous groups at baseline, we were able to address the male/female difference by employing ANCOVA adjusting for sex during the data analysis phase.

## 5. Conclusions

The present study reveals that a dosage of 6 mg cafestol twice daily for a period of twelve weeks causes reductions in visceral fat content, body weight, and gamma-glutamyl transferase levels compared to placebo. However, cafestol does not lead to improvements in insulin sensitivity or glucose tolerance among participants with an increased waist circumference. This does not exclude positive effects on insulin sensitivity and glucose tolerance with higher cafestol dosages or longer treatment duration. It would be recommendable to conduct these studies in populations with more pronounced insulin resistance and/or individuals diagnosed with type 2 diabetes. Cafestol may serve as a potential substance for weight and visceral fat reduction in the future.

## Figures and Tables

**Table 1:** Baseline data for the 40 participants. Baseline data. Data are listed as mean (SD) or as numbers of participants (N (%)).

Data—Mean (SD)		Cafestol (N = 20)	Placebo (N = 20)	Total (N = 40)
	Age (years)	60.2 (15.7)	57.2 (10.2)	58.7 (13.2)
	Weight (kg)	96.7 (11.7)	94.1 (15.3)	95.4 (13.5)
	BMI (kg/m^2^)	32.7 (3.5)	31.6 (3.1)	32.1 (3.3)
Coffee intake (cups/day)	Total	3.5 (2.8)	5.3 (3.6)	4.4 (3.3)
	With cafestol	1.3 (2.3)	2.4 (3.2)	1.9 (2.8)
Waist circumference (cm)	Male	114.4 (6.1)	112.1 (6.1)	113.5 (6.0)
	Female	104.6 (8.8)	102.3 (8.3)	103.2 (8.3)
	All participants	110.6 (8.5)	106.1 (8.8)	108.3 (8.9)
IST Glucose (mmol/L)	Start	8.1 (1.9)	7.8 (2.3)	7.9 (2.1)
	Stage 1 SSPG	6.6 (0.9)	6.7 (0.9)	6.7 (0.9)
	Stage 2 SSPG	10.4 (3.9)	9.7 (4.8)	10.0 (4.3)
Mixed Meal	Glucose tAUC (mmol/L × 240 min)	1427 (134)	1438 (168)	1432 (150)
CGM	Mean glucose (mmol/L)	5.8 (0.5)	5.9 (0.5)	5.9 (0.5)
	Time in Range (%)	96 (8)	98 (2)	97
	Glucose variability (24 h-standard deviation)	1.0 (0.3)	1.0 (0.2)	1.0 (0.2)
MRI	Liver fat %	8.2 (8.2)	8.6 (8.8)	8.4 (8.4)
	Visceral fat (L)	7.9 (2.8)	7.0 (2.6)	7.4 (2.7)
	Subcutaneous fat (L)	13.9 (4.7)	13.8 (4.0)	13.8 (4.3)
24-h blood pressure (mmHg)	Systolic BP (awake)	129 (12)	133 (15)	131 (14)
	Diastolic BP (awake)	76 (6)	82 (7)	79 (7)
	Systolic BP (asleep)	114 (15)	115 (20)	114 (18)
	Diastolic BP (asleep)	64 (5)	67 (12)	66 (9)
Fasting blood samples	HbA1c (%, mmol/mol)	5.5%, 36.6 (3.1)	5.4%, 35.5 (3.1)	5.5%, 36.1 (3.1)
	HOMA-IR (C-peptide)	43.1 (16.1)	40.1 (10.8)	41.6 (13.6)
	HOMA-IR (Insulin)	4.2 (2.4)	4.0 (2.2)	4.1 (2.3)
	Cholesterol (mmol/L)	4.6 (1.0)	5.1 (1.3)	4.8 (1.1)
	HDL-cholesterol (mmol/L)	1.1 (0.3)	1.3 (0.4)	1.2 (0.4)
	LDL-cholesterol (mmol/L)	2.9 (0.7)	2.9 (0.8)	2.9 (0.8)
	Triglycerides (mmol/L)	1.3 (0.5)	1.6 (1.0)	1.4 (0.8)
Data—N (%)				
Sex	Male	11 (55%)	7 (35%)	18 (45%)
	Female	9 (45%)	13 (65%)	22 (55%)
	Cholesterol-Lowering drug use	6 (30%)	4 (20%)	10 (25%)
	Anti-Hypertensive drug use	4 (20%)	4 (20%)	8 (20%)
	Family history of type 2 diabetes	7 (35%)	7 (35%)	14 (35%)

**Table 2:** Primary and secondary outcomes in 20 subjects receiving cafestol and 20 subjects receiving placebo.

	Group difference			
	Absolute β_cafestol_	Relative β_cafestol_	95%CI	*p*
Insulin Suppression Test				
Stage 1 SSPG (mmol/L)	0.18		−0.21–0.56	0.4
Stage 2 SSPG (mmol/L)	0.44		−1.14–2.02	0.6
Mixed Meal Test				
Glucose tAUC (mmol/L × 240 min)	36.6		−22.1–95.2	0.22
Insulin tAUC (pmol/L × 240 min)	−4923		−12,154–2307	0.18
Glucagon tAUC (pmol/L × 240 min)	−319		−747–108	0.14
Triglyceride tAUC (mmol/L × 240 min)		0.98	0.84–1.14	0.8
MRI				
Liver fat %	−0.14		−2.2–2.0	0.9
Visceral fat (L)	−0.39		−0.7–−0.1	0.014 *
Subcutaneous fat (L)	0.08		−0.7–0.9	0.8
24 h Blood Pressure (mmHg)				
Daytime systolic ^†^	−5.1		−13–3	0.20
Daytime diastolic ^†^	−2.2		−7–3	0.4
Nighttime systolic ^‡^	−1.4		−12–9	0.8
Nighttime diastolic ^‡^	−2.0		−9–5	0.6
Continuous Glucose Monitor				
Mean Glucose (mmol/L) ^§^		1.01	0.96–1.07	0.6
Time in Range (%) ^§^	0.6		−0.8–2.1	0.4
Glucose Variability ^§^	0.03		−0.08–0.15	0.6
Fasting Blood samples				
HbA1c (mmol/mol)		0.96	0.95–1.02	0.5

Primary and secondary outcomes. ANCOVA results with the absolute effect of cafestol (absolute β_cafestol_), the relative effect of cafestol when appropriate (Relative β_cafestol_), 95% CI, and ANCOVA *p*-value. The ANCOVA model is adjusted for baseline values and sex. * represents ANCOVA *p*-value < 0.05. Numbers analyzed: 20/20 in both groups except ^†^ cafestol group 19/20, ^‡^ cafestol group 12/20, ^‡^ placebo group 10/20, and ^§^ placebo group 19/20.

**Table 3:** Fasting blood samples and ancillary analyses of 20 subjects receiving cafestol and 20 subjects receiving placebo.

	Group Difference			
	Absolute β_cafestol_	Relative β_cafestol_	95%CI	*p*
Fasting Blood samples				
LDL Cholesterol (mmol/L) ^†^	−0.06		−0.30–0.18	0.6
HDL Cholesterol (mmol/L)	0.03		−0.10–0.15	0.7
Plasma Glucose (mmol/L)	0.08		−0.11–0.26	0.4
Total Cholesterol (mmol/L)	−0.06		−0.38–0.26	0.7
Triglycerides (mmol/L)		0.98	0.81–1.19	0.9
Alanine Aminotransferase (U/L)		0.97	0.81–1.16	0.7
Aspartate Aminotransferase (U/L) ^‡^		1.07	0.96–1.19	0.24
Gamma-Glutamyltransferase (U/L)		0.85	0.73–0.97	0.019 *
Creatinine (μmol/L)	2.90		−0.48–6.28	0.09
High-sensitivity CRP (mg/L)		1.03	0.56–1.92	0.9
HOMA-IR (Insulin)		0.99	0.77–1.27	0.9
HOMA-IR (C-peptide)	0.71		−4.16–5.58	0.8
Ancillary analyses: Insulin Suppression Test				
Stage 1 FFA (mmol/L)	0.07		0.00–0.14	0.048 *
Ancillary analyses: MRI				
Gluteus muscle fat %	−0.22		−2.0–1.6	0.8
Psoas major muscle fat %	0.09		−0.9–1.1	0.9
Ancillary analyses: Weight & Height				
Weight (kg)	−1.79		−3.45–−0.13	0.035 *
BMI (kg/m^2^)	−0.59		−1.16–−0.02	0.043 *

Fasting blood samples and additional ancillary outcome measures. ANCOVA results with the absolute effect of cafestol (absolute β_cafestol_), the relative effect of cafestol when appropriate (relative β_cafestol_), 95% CI, and ANCOVA *p*-value. The ANCOVA model is adjusted for baseline values and sex. * represents ANCOVA *p*-value < 0.05. Numbers analyzed: 20/20 in both groups except ^†^ placebo group 18/20, ^‡^ cafestol group 19/20, and ^‡^ placebo group 19/20.

## Data Availability

The datasets generated during and/or analyzed in the current study are available from the corresponding author upon reasonable request.

## Supplementary Materials

The following supporting information can be downloaded at: https://www.mdpi.com/article/10.3390/nu16193232/s1, Table S1: Baseline and post-interventional results in 20 subjects receiving cafestol and 20 subjects receiving placebo before and after intervention. CONSORT 2010 checklist of information to include when reporting a randomised trial.

## Appendix A. Statistical Analysis

All statistical analyses were performed in R using R-Studio (RStudio Team (2020). RStudio: Integrated Development for R. RStudio, PBC, Boston, MA, USA. URL http://www.rstudio.com/, accessed on 15 August 2024).

Total AUCs were calculated using the auc() function from the MESS package with the trapezoid method.

Glucose levels at IST start and at steady state (Stage 1 and Stage 2), FFA, glucagon and insulin were calculated individually for each participant on both ISTs separately as participant-mean levels at time points −15 and 0 min, 100, 110 and 120 min and 220, 230 and 240 min, respectively.

ABPM recordings were analyzed and split into systolic and diastolic blood pressure during daytime (awake) and nighttime (asleep).

MRI spectra were analyzed using LCModel version 6.3-1L. MRI Dixon fat content was assessed using a home-made Windows program (Siswin).

CGM data were analyzed, and a mean glucose and standard deviation was calculated for each participant every day. Data from each participant’s first and last day of CGM monitoring was excluded, so that all days analyzed had 24-h data. A mean-mean glucose was calculated for each participant before and during the last weeks of intervention, along with a mean-standard-deviation glucose as a measure of glucose variability.

The results were grouped by treatment and visit (before and after intervention) and the results are presented in the Supplementary Table S1 as mean or median (SD). Normality was assessed using the Shapiro Wilks test, and if the results in a treatment group (before and after intervention) could reasonably be assumed to be normally distributed (Shapiro Wilks test *p* > 0.05), a paired t-test was used to compare within-group mean difference. In cases where the results were not normally distributed (Shapiro Wilks test *p* < 0.05), Wilcoxon signed rank tests were used to compare within-group difference, and medians (and median differences) are shown. The study employed a one-way ANCOVA to examine the impact of treatment (independent variable, cafestol or placebo) on post-intervention outcomes. The glm() function from the r stats package was used, from which βcafestol denoted the difference between treatment groups on post-intervention results, adjusted for pre-intervention results and sex. Interactions between treatment group and pre-intervention levels were assessed. Residuals were evaluated for normality using the Shapiro Wilk test, where a *p*-value greater than 0.05 indicated normally distributed residuals. Levene’s test was used to assess the equal residual variance in both groups, where a *p*-value greater than 0.05 suggested homogeneity of the residual variances. In cases where residuals were not normally distributed and/or did not have equal variance, and visual inspection of plots confirmed that the ANCOVA assumptions were not met, an ANCOVA was performed on log-transformed data again using the glm() function, where exp(βcafestol) represented the relative difference between groups. In instances where post-intervention data was missing, the corresponding pre-intervention measurement from the same participant was removed, and vice versa.
